# Characterising the pharmacokinetics of olanzapine in patients with anorexia nervosa (TORPEDO): protocol for a multicentre cross-sectional study

**DOI:** 10.1136/bmjopen-2026-119486

**Published:** 2026-09-15

**Authors:** Jiayi Liang, Bram Dierckx, Gwen C Dieleman, Tessa M Bosch, Birgit C P Koch, Brenda C M De Winter

**Affiliations:** 1 Department of Hospital Pharmacy, Erasmus MC University Medical Center Rotterdam, Rotterdam, The Netherlands; 2 Rotterdam Clinical Pharmacometrics Group, Erasmus MC University Medical Center Rotterdam, Rotterdam, The Netherlands; 3 Department of Child and Adolescent Psychiatry and Psychology, Erasmus MC University Medical Center Rotterdam, Rotterdam, The Netherlands; 4 Department of Clinical Pharmacology & Toxicology, Maasstad Laboratory, Maasstad Hospital, Rotterdam, The Netherlands; 5 Department of Hospital Pharmacy, Maasstad Hospital, Rotterdam, The Netherlands

**Keywords:** Adolescents, PSYCHIATRY, Pharmacists, Adolescent, Adult psychiatry

## Abstract

**Abstract:**

**Introduction:**

Anorexia nervosa (AN) is a debilitating eating disorder with one of the highest mortality rates of all psychiatric disorders. The lifetime prevalence rates of AN might be up to 4% among females and 0.3% among males. According to a systematic review, the pooled incidence rate of AN in females is 8.8 per 100 000 person-years; 95% CI 7.8 to 9.8. The path towards full recovery is often long and only half of the patients recover completely after 10 years. As part of the treatment of AN, olanzapine is frequently used to reduce severe anxiety, agitation and obsessive compulsions. Due to a dearth of pharmacokinetic studies on olanzapine in this population with altered body composition, and side effects, doctors often dose low out of precaution. These low doses are potentially subtherapeutic and elongate the progression and the treatment. In this study, we aim to research the pharmacokinetics of olanzapine in AN patients to facilitate more adequate dosing strategies in this vulnerable group.

**Methods and analysis:**

Towards precision dosing of olanzapine in anorexia nervosa patient**s** (TORPEDO) is a multicentre, cross-sectional observational study. It includes patients aged 12–30 years who are being treated with olanzapine for either AN (n=30) or other non-AN psychiatric indications (n=30). The aim is to develop population pharmacokinetic models of patients with AN and those without, in order to compare how AN affects the pharmacokinetics of olanzapine. Olanzapine plasma day-curve will be obtained using capillary micro sampling. In a secondary analysis, we will examine associations between olanzapine pharmacokinetic parameters and clinical outcomes, including treatment effectiveness, extrapyramidal side effects, cardiac abnormalities and metabolic laboratory markers.

**Ethics and dissemination:**

This study was approved by the Medical Ethics Committee of the Erasmus Medical Center in Rotterdam, the Netherlands (registration number MEC-2022-0687). This ethical approval covers all study sites. Every significant amendment to the protocol will have to be approved by the medical ethics committee. The local feasibility was tested by the medical ethics committee and board of directors of all study sites included in the study. Written informed consent will be obtained from the patients and/or the legal representative(s) before enrolment in the study. Findings will be disseminated through publication in peer-reviewed journals and presentation at national and international conferences. Results will contribute to the evidence base for optimising olanzapine dosing in patients with AN.

**STRENGTHS AND LIMITATIONS OF THIS STUDY**

- Prospective study with multiple data points collected from the same individual.
- Parametric power estimation was performed to determine the required sample size.
- The self-sampling blood collection method may introduce bias in the measured concentrations.

## Introduction

 Anorexia nervosa (AN) is a debilitating psychiatric disorder that often emerges during adolescence.^1–4^ The disorder is marked by extreme calorie restriction, an inability to maintain a healthy body weight (often falling below 85% of their ideal weight), an overwhelming fear of gaining weight and a distorted perception of their body image.^5^ Consequently, AN is associated with one of the highest mortality rates of any psychiatric disorder, owing to both the life-threatening physiological effects of chronic malnutrition and an increased risk of suicide.^3^ Treating AN is a challenging, long-term process, primarily relying on nutritional advice, psychotherapy and family counselling.^6^ Unfortunately, only about half of the patients fully recover within 4–10 years.^7^ For patients with treatment-resistant AN, antipsychotics may be considered as adjunctive treatment options when delivered on an individualised basis and under specialist multidisciplinary supervision. Reflecting their use in routine clinical practice, an observational study of 525 individuals with AN reported that approximately 13% were receiving antipsychotic medication; however, the proportion of these patients with treatment-resistant AN was not reported.^8^

Among all antipsychotics, olanzapine demonstrated the strongest evidence of effectiveness in the treatment of AN, most notably in promoting increases in body mass index (BMI) over time.^9^ Olanzapine is employed to reduce anxiety and agitation and mitigate obsessions or delusions regarding weight and body shape.^10 11^ National and international guidelines agree that the use of low-dose olanzapine in acute-phase treatment of patients with severe and enduring AN is justified in individual cases.^12^

Despite evidence supporting the efficacy of olanzapine in treating AN and its association with increased body weight in adults, its effect in adolescents remains unclear.^13^ Moreover, an ongoing challenge in treating AN patients—especially those with altered body composition and potentially altered pharmacokinetics—is determining the optimal dosage. To date, no studies have explored the pharmacokinetic properties of any psychotropic medications, including olanzapine, in individuals with extreme low body weights. In cases of cachexia from other causes, changes in absorption, distribution and metabolism have been documented.^14^ In AN, however, only absorption has been studied for substances such as zinc, mannitol and lactulose and found to be reduced.^15 16^ These findings suggest that reduced absorption in AN patients could lead to lower plasma levels and, consequently, decreased efficacy. Although evidence linking olanzapine concentrations to treatment response in AN remains limited, higher doses have been associated with greater clinical benefit.^17^ However, this observation is based on pooled heterogeneous studies rather than formal concentration-response analyses. Conversely, an altered body composition resulting in reduced volume of distribution and impaired metabolism in these patients may result in higher drug exposure, increasing the risk of side effects such as extrapyramidal effects, which can cause significant discomfort and potentially irreversible sequelae.^18 19^ Additionally, olanzapine has been shown to cause serious cardiac arrhythmias by prolonging the QT interval, which may exacerbate the already elevated risk of cardiac events—such as ventricular tachycardia, aborted cardiac arrest or full cardiac arrest—in patients with AN.^20^ Furthermore, olanzapine is associated with a high rate of metabolic disturbances and can contribute to the development of diabetes. Studies have shown that the combination of elevated cholesterol levels^21 22^ and diabetes mellitus in patients with AN is associated with a nearly 40% mortality rate over 10 years.^23^

In view of olanzapine’s many side effects, clinicians often adopt a ‘start low, stay low’ dosing strategy, particularly in patients with the lowest BMI. For these individuals, such an approach may result in subtherapeutic doses, potentially worsening their prognosis. Supporting this hypothesis, studies involving patients treated with olanzapine for conditions other than AN have shown that over one-third of patients deemed treatment-resistant to antipsychotics had subtherapeutic plasma levels of olanzapine.^24^ These levels were linked to poorer treatment outcomes and more frequent hospital admissions, leading to a longer course of treatment and higher healthcare costs. Given these insights, we believe it is crucial to develop tailored dosing guidelines for using olanzapine in patients with AN.

The current study aims to improve the treatment of AN patients by optimising olanzapine dosing. We will achieve this by exploring the pharmacokinetics of olanzapine in these patients and comparing these results with those of non-AN individuals. Based on the population pharmacokinetic model, we will obtain vital information on relationships between plasma concentrations, efficacy and side effects. These data will refine dosing strategies and avoid subtherapeutic exposure ultimately improving the effectiveness and safety of olanzapine in this highly vulnerable population—patients with AN.

## Methods and analysis

TORPEDO is a national, multicentre, cross-sectional cohort study designed to investigate the pharmacokinetics of olanzapine in patients with AN. In the Netherlands, participants will be recruited from five specialised psychiatric centres and two academic medical centres. In individuals treated with oral formulated olanzapine, drug exposure will be systematically assessed. The study design does not involve longitudinal follow-up, as all procedures and measurements are completed during a 1 day study visit. The study population will include patients diagnosed with AN as well as those with other non-AN psychiatric conditions. Pharmacokinetic models will be developed using Nonlinear Mixed Effects Modelling (NONMEM) analysis. An overview of the study timeline is presented in online supplemental material. The study protocol was approved by the Medical Ethics Committee of the Erasmus Medical Center in Rotterdam on 1 October 2022. The current protocol is V.5, dated February 2025. No clinical trial registration number is applicable. The study is ongoing and has not yet been completed. Participant inclusion started on 21 November 2023. Recruitment is ongoing, and the study is currently in the inclusion phase.

### Study procedure

Participants are screened for eligibility based on the study’s inclusion and exclusion criteria. Those who meet the eligibility requirements and provide written informed consent are enrolled in the study. Following enrolment, retrospective data are collected from available records. Participants subsequently complete dried blood spot (DBS) day-curve sampling and self-report questionnaires as part of the study assessments.

### Participants

Both inpatients and outpatients at participating centres are eligible for inclusion if they are between 12 and 30 years of age. Inclusion was restricted to individuals aged 12–30 years to minimise age-related variability and facilitate assessment of the effects of AN on olanzapine pharmacokinetics. The AN group must have a documented clinical diagnosis based on Diagnostic and Statistical Manual of Mental Disorders (DSM)-IV or DSM-V criteria. The reference group must have a diagnosed psychiatric disorder other than AN. All patients must be on a stable regimen of orally administered olanzapine, have achieved steady-state exposure, and have had no missed doses during the previous 7 days. Each group will include a total of 30 participants. Exclusion criteria are: (1) concurrent use of carbamazepine, lopinavir, rifampicin, ritonavir, ciprofloxacin and fluvoxamine, (2) presence of congenital or acquired syndrome associated with changes in appetite, body weight or lipid profile (eg, Prader Willi), and (3) pregnancy.

### Informed consent

According to Dutch law and the Declaration of Helsinki, for patients aged 12 to 16 years, informed consent must be obtained from both the patient and their parent(s) or legal representative. For patients aged 16 or older, only the patient needs to sign for informed consent. Informed consent will be obtained by the researcher or an independent research nurse. Participants can withdraw from the study at any time, without having to state a reason and without any consequences. The investigator can decide to withdraw a subject from the study for urgent medical reasons.

### Sample size calculation

To determine the required sample size, we employed a simulation-based approach. A parametric bootstrap was performed using Stochastic Simulation and Estimations for study design evaluation and power analysis.

As a starting point, we used a published pharmacokinetic model of olanzapine in paediatric patients aged 2 months to 19 years.^25^ Given the high interindividual variability observed in this population (0.309) compared with adults (0.091),^26^ we adjusted the variability to 0.200 to more accurately reflect our target population of adolescents and young adults. To account for the hypothesised impaired hepatic clearance in AN patients, we incorporated a 30% reduction in clearance, based on findings by Trobec *et al* in cachectic patients.^14^ The specific pharmacokinetic model parameters used in the simulations are summarised in table 1.

**Table 1:** Population pharmacokinetic model of olanzapine used for power calculation

Parameter	Estimate
Ka (1 /h)	0.758
CL/F (L/h, 70 kg)	16.8
V/F (L, 70 kg)	663
θ_CL,WT_	0.486
θ_CL,AN_	0.7
ω_CL/F_^2^	0.200
Random effects	
ε_1_^2^ – proportional error	0.0772
ε_2_^2^ – additional error	1.51

CL/F, apparent clearance ($CL/F_{i}\left(L∙h^{-1}\right)=16.8*(\frac{WT_{i}}{70kg}{)}^{0.486}*\frac{{PMA_{i}}^{3.97}}{70^{3.97}+{PMA_{i}}^{3.97}}*exp(\eta_{i,\frac{CL}{F}})$); Ka, absorption rate constant ($K_{a}\left(h^{-1}\right)=0.758$); V/F, apparent volume of distribution ($V/F_{i}\left(L\right)=663*(\frac{WT_{i}}{70kg}{)}^{1}$); θ_CL, WT_, exponent modulation of the influence of weight on apparent clearance.

Next, the model was used to simulate the olanzapine concentration–time profiles in 50 virtual patients, with body weights ranging from 31 to 54 kg and daily olanzapine doses between 2.5 and 12.5 mg. For each simulated individual, hourly plasma concentrations over a 24-hour period were generated. Pharmacokinetic parameters were then re-estimated using only 1–3 samples taken between 2 and 23 hours postdose. These limited-sample estimates were compared with those derived from the full dataset to assess accuracy. Optimal sampling time points had been identified at 4, 8 and 23 hours postdose. Optimal sampling time points were identified at 4, 8 and 23 hours following dose administration. In clinical practice, blood samples will be collected as close as possible to these target time points while minimising disruption to patients’ daily routines, including sleep. Sampling schedules will therefore be individualised based on the dosing regimen and determined in consultation with each patient. To ensure accurate pharmacokinetic assessment, patients will be instructed to record the exact times of both dose administration and blood sample collection.

**Figure 1:**
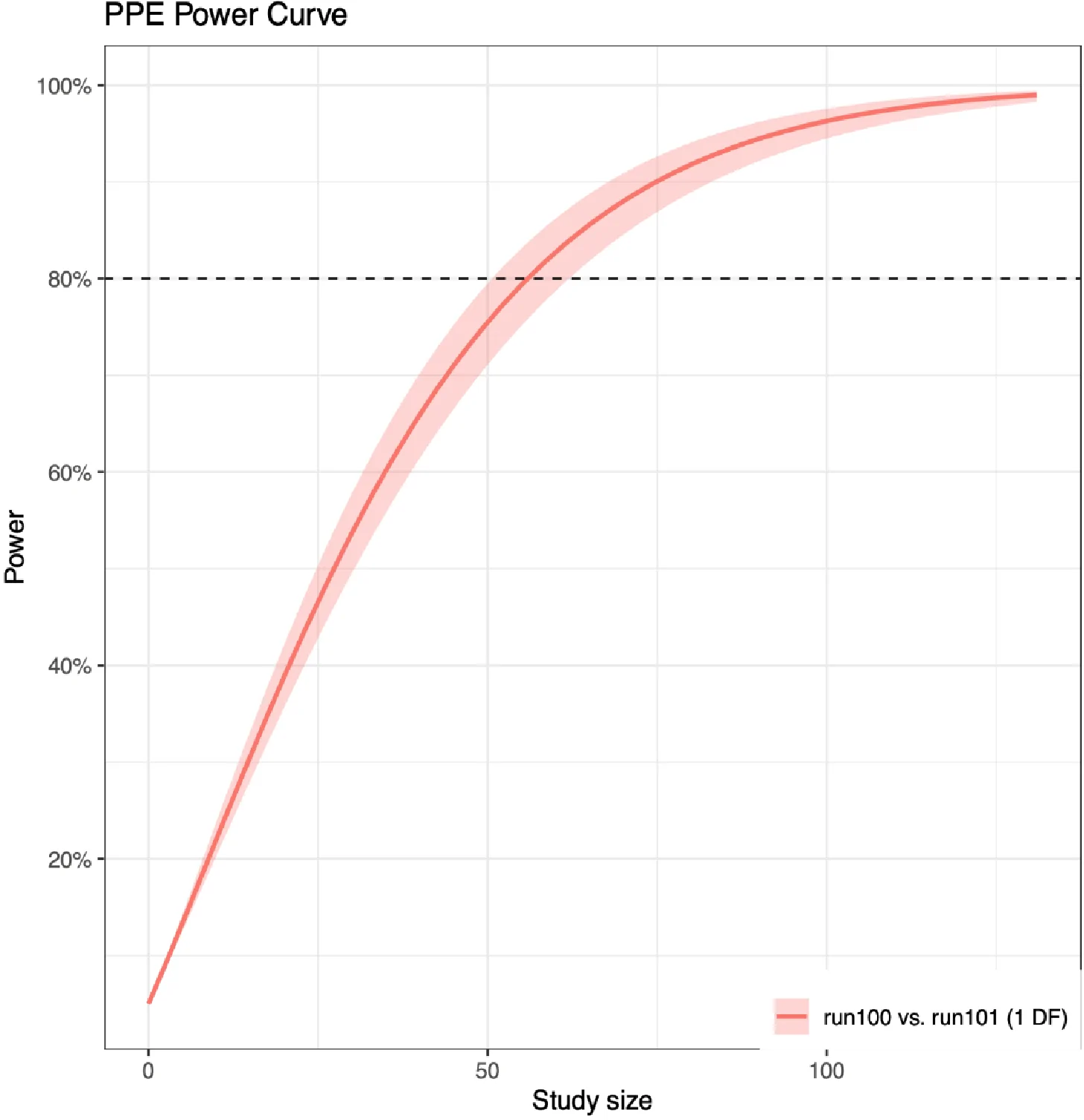
Parametric power estimation (PPE) power curve, sample size calculation.

To evaluate the model’s ability to detect a 30% reduction in clearance associated with AN, beyond the effect of body weight, that may influence drug disposition and pharmacokinetic parameters independently of body size we simulated and re-estimated data using both the original model (including the AN effect on clearance) and an alternative model without this effect. Each model was run 100 times using a virtual cohort of 100 patients (50 with AN and 50 without). Based on these simulations, a sample size of approximately 56 participants (28 with AN and 28 without) was required to achieve 80% statistical power, as illustrated in figure 1. Allowing for a 5% drop-out rate, we aimed to include a total of 60 patients.

### Pharmacokinetic sampling

Blood sampling for olanzapine quantification will primarily be performed using the DBS method, where blood is collected via a fingerprick. A few drops of blood will be placed onto Whatman 903 filter paper. This method has been successfully validated for olanzapine quantification in whole blood (ref: Capillary olanzapine sampling method validation study (COSMOS) study, *unpublished*). Microsamples will be collected at three different time points throughout the day. In addition to DBS, residual blood samples obtained via venipuncture on the same day will also be collected, if available. Serum will be extracted from these samples and analysed for olanzapine concentration.

### Study parameters and assessments

The following text outlines the study outcomes and corresponding assessments. An overview of all assessments and their respective time points is provided in table 2. Measures marked with **X** are not typically part of standard clinical care in most centres, whereas those marked with **√** represent procedures that are routinely performed as part of standard care.

**Table 2:** Measurements and monitoring

Assessment	Baseline (retrospective data collection)	At inclusion
**Primary outcome**		
Weight, height and BMI	**√**	**√**
Olanzapine levels	NA	X
**Secondary outcome**		
Lab-workup*	**√**	**√**
ECG	X	
Vital parameters	**√**	**√**
EDE-Q	**√**	**√**
EDI-3	**√**	**√**
BDI-II-NL-R	X	X
CY-BOCS/Y-BOCS	X	X
SCARED-NL	X	X
BAI-NL	X	X
ESS	X	X
SAS	X	**X**
**Others**		
DNA-sampling		X

EDE-Q (>12 year): The Eating Disorder Examination Questionnaire is a 28-item self-reported questionnaire to assess the range and severity of features associated with a diagnosis of an eating disorder using four subscales (Restraint, Eating Concern, Shape Concern and Weight Concern) and a global score.

BDI-II-NL-R (>13 year): Beck depression inventory-II is a 21-item self-reported questionnaire to assess the severity of depression in adults and adolescents.

(C)Y-BOCS (8–18 year and >18 year): Yale-Brown Obsessive Compulsive disorder is a semi-structured interview considered to be the gold standard in the measurement of obsessive-compulsive disorder (OCD) severity.

SCARED-NL (7–19 year): Screen for Child Anxiety Related Emotional Disorders is 69-item self-reported questionnaire to assess the symptoms of anxiety in children and adolescents.

BAI-NL (>17 year): Beck anxiety inventory-II is a 21-item self-reported questionnaire to assess the severity of anxiety in adults and adolescents.

EDI-3 (13–39 year): The Eating Disorder Inventory 3 is a standardised clinical evaluation of symptomatology associated with eating disorders. It provides data of the frequency of symptoms (ie, binge eating, self-induced vomiting, exercise patterns, use of laxatives, diet pills and diuretics).

SAS: The Simpson Angus Scale is a performance scale that measures drug-induced parkinsonism symptoms.

DNA-sampling: whole genome sequencing.

* Cholesterol, lipoproteins, glucose, liver parameters, renal function, albumin.

BMI, body mass index.

#### Primary outcome

We will develop a pharmacokinetic model of olanzapine incorporating parameters such as the absorption rate constant (Ka), clearance (CL) and volume of distribution (Vd). Within this model, we will investigate the effects of AN and body weight on these pharmacokinetic parameters. The primary endpoint of this analysis is to evaluate the impact of AN on CL. This will allow us to compare pharmacokinetic parameters between AN and non-anorexic patients in order to optimise precision dosing in the AN population.

#### Secondary outcomes

Secondary, we will use this model to simulate the exposure such as area under the curve (AUC), peak plasma concentration (Cmax) and time to peak plasma concentration (Tmax). The exposure will be linked to safety parameters (eg, ECG alteration, sedation, extrapyramidal symptoms and metabolic changes) and the efficacy data in a pharmacodynamics analysis.

*Safety parameters* include the metabolic side effects such as blood glucose, cholesterol, lipoproteins and triglyceride levels. ECG changes, if available, will also be documented. Neuroleptic-induced parkinsonism symptoms will be evaluated by the treating physicians using the Simpson-Angus Scale (SAS), a clinical tool for assessing drug-induced parkinsonism. Sedation related to olanzapine will be measured using the Epworth Sleepiness Scale (ESS), which estimates the presence of excessive daytime sleepiness that may warrant medical attention. Lastly, blood pressure and heart rate will be measured at the study visit.

*Effectivity* is defined as the score of the self-reporting and interview-based questionnaires, consisting of the following:

- Eating disorder: Eating Disorder Examination Questionnaire (EDE-Q), a 28-item self-reported questionnaire to assess the range and severity of features associated with a diagnosis of an eating disorder using four subscales (Restraint, Eating Concern, Shape Concern and Weight Concern) and a global score. And Eating Disorder Inventory 3 (EDI-3), a standardised clinical evaluation of symptomatology associated with eating disorders. It provides data of the frequency of symptoms (ie, binge eating, self-induced vomiting, exercise patterns, use of laxatives, diet pills and diuretics).
- Depression: Beck depression inventory-II (BDI-II-NL-R), a 21-item self-reported questionnaire to assess the severity of depression in adults and adolescents.
- Anxiety disorder: Screen for Child Anxiety Related Emotional Disorders (SCARED-NL), a 69-item self-reported questionnaire to assess the symptoms of anxiety in children and adolescents, and Beck anxiety inventory-II (BAI-NL), a 21-item self-reported questionnaire to assess the severity of anxiety in adults and adolescents.
- Obsessive compulsive disorder: Yale-Brown Obsessive Compulsive disorder semi-structured interview ((C)Y-BOCS), a semi-structured interview considered to be the gold standard in the measurement of obsessive-compulsive disorder (OCD) severity.

*Other study parameters and assessments* consist of DNA sampling by buccal swab to determine CYP1A2, CYP2D6 and UGT1A1 metaboliser status, which will be investigated as potential moderators for the pharmacokinetics.

### Statistical analyses

#### Primary outcome

A population pharmacokinetic model for AN-patients and non-AN-patients will be developed using a population pharmacokinetic modelling approach using NONMEM V.7.4.4 (FOCE+I; ICON Development Solutions, Ellicott City, MD, USA) and PsN V.4.7.0. Pirana software V.2.9.7 was used as an interface between NONMEM and R (V.4.1.3).

The values for Vd and CL will be estimated. In addition, the AUC, Tmax and the Cmax will be calculated. Between-subject variability (BSV) will be assessed for each pharmacokinetic parameter using an exponential model. Residual variability was evaluated using an additive, proportional or combined error model. Second, correlations between pharmacokinetic parameters and potential covariates (effect of AN, age, gender, weight, BMI, BMI z-score, pharmacogenetic profile, laboratory data (like renal function, liver function, albumin) and comedication) will be tested using forward inclusion and backward elimination. To correct for multiple covariates, a lower p-value of respectively <0.05 and <0.01 will be considered to be significant. Minimum objective function values, parameter precision, residual error estimates and visual inspection of the goodness-of-fit plots were considered for model selection. The final model will be validated using bootstrap with resampling (n=1000), visual predictive checks (VPCs) and VPCs stratified for covariates (n=1000).

#### Secondary outcomes

In the pharmacodynamic analysis, we will assess the relationships between olanzapine’s AUC, Cmax and Tmax and both treatment effectiveness—measured using the EDE-Q, BDI-II-NL-R, SCARED-NL, and (C)Y-BOCS scores—and side effects, including those assessed by the SAS, ESS, ECG and metabolic changes (eg, blood pressure, cholesterol and lipoprotein levels). These relationships will be evaluated using Pearson or Spearman correlation coefficients, as appropriate, and diagnostic performance will be assessed using Receiver Operating Characteristic (ROC) analysis. All analyses will be conducted in RStudio (V.2023.06.1; RStudio, PBC, Boston, MA, USA) running R V.4.3.1. A p value <0.05 will be considered statistically significant.

### Data handling and monitoring

Questionnaires will be disseminated through an online server and health data collected by researchers will be stored in an electronic case report form. Patient data will be pseudo-anonymised through allocation of subject identification codes. Data will be stored on a secure drive with limited access. Paper data will be stored in locked filing cabinets. Data handling will be in line with the General Data Protection Regulation.

Because this study has a low risk of low complications, a monitor will visit each study site every 12 months. 25% of all cases are randomly selected for verification by the independent monitor. Informed consent, source data and reported serious adverse events (SAEs) are reviewed for errors.

### Serious adverse events

SAEs considered related to the monitoring methods will be reported to the local medical ethics committee through an online platform within 7 days of first knowledge for SAEs that result in death or are life threatening. All other SAEs will be reported within a period of maximum 15 days after the sponsor has first knowledge of the SAEs.

### Patient and public involvement

None.

## Ethics and dissemination

This study was approved by the Medical Ethics Committee of the Erasmus Medical Center in Rotterdam, the Netherlands (registration number MEC-2022-0687). This ethical approval covers all study sites. Every significant amendment to the protocol will have to be approved by the medical ethics committee. The local feasibility was tested by the medical ethics committee and board of directors of all study sites included in the study. Written informed consent will be obtained from the patients and/or the legal representative(s) before enrolment in the study. Findings will be disseminated through publication in peer-reviewed journals and presentation at national and international conferences. Results will contribute to the evidence base for optimising olanzapine dosing in patients with AN.

## Discussion

In the complex treatment of AN, olanzapine has shown potential effectiveness in alleviating symptoms. However, current pharmacokinetic knowledge lacks the foundational data needed to support informed and individualised dosing in this vulnerable population. The TORPEDO study will be the first pharmacokinetic study conducted specifically in individuals with AN.

A major strength of this study is its prospective design, with multiple pharmacokinetic data points collected from the same individual, enabling a detailed characterisation of olanzapine exposure. Furthermore, a parametric power estimation was performed to determine the required sample size, ensuring that the study is adequately powered to achieve its primary objectives.

Through our strong collaboration within the research consortium established by the Safety and Pharmacokinetics of Antipsychotics in Children with autism and Safety and pharmacokinetics of antipsychotics in children 2 (SPACe and SPACe2:STAR) study, we have developed a robust network of professionals committed to supporting the TORPEDO study—particularly in overcoming one of its greatest challenges: the recruitment of individuals with AN. To facilitate this, we have engaged a wide network of specialised treatment centres for eating disorders, thereby expanding our reach and enhancing recruitment potential.

A potential limitation of the study is the use of a self-sampling blood collection method, which may introduce variability and bias in the measured olanzapine concentrations. In addition, the cross-sectional nature of the study limits the ability to assess longer-term relationships between drug exposure, treatment response and adverse effects. To draw robust conclusions from the secondary endpoints, long-term follow-up of clinical parameters would ideally be required. Therefore, we plan to conduct a prospective follow-up study in the future to address this gap.

In conclusion, the TORPEDO study is a unique and pioneering study. Its findings will be the first to offer pharmacokinetic insights specific to the anorectic population, shedding light on the relationships between plasma concentrations, therapeutic efficacy and side effects. By developing a population pharmacokinetic model, this study will provide essential data to guide clinicians in making more informed and precise dosing decisions—ultimately improving the safety and effectiveness of olanzapine treatment in patients with AN.

## Supplementary material

10.1136/bmjopen-2026-119486 online supplemental file 1
