# Supplementary material for: Characterising the pharmacokinetics of olanzapine in patients with anorexia nervosa (TORPEDO): protocol for a multicentre cross-sectional study
Source: BMJ Open. 2026 Sep 15;16(9):e119486. doi: 10.1136/bmjopen-2026-119486 (PMC13583754; doi:10.1136/bmjopen-2026-119486)
Supplement: Supplementary file 1 [file bmjopen-2026-119486.reviewer_comments.pdf]

PEER REVIEW HISTORY

BMJ Open publishes all reviews undertaken for accepted manuscripts. Reviewers are asked to complete a checklist review form and are provided with free text boxes to elaborate on their assessment. These free text comments are reproduced below.

ARTICLE DETAILS

Title (Provisional)

Characterising the pharmacokinetics of olanzapine in patients with anorexia nervosa (TORPEDO): protocol for a multicentre cross-sectional study

Authors

Liang, Jiayi; Dierckx, Bram; Dieleman, Gwen C.; Bosch, Tessa M.; Koch, Birgit C. P.; De Winter, Brenda C.M.

VERSION 1 - REVIEW

**Reviewer**	**1**
**Name**	**O'Neill, James**
**Affiliation**	**University of Leeds School of Medicine**
**Date**	**29-Jun-2026**
**COI**	**I am a member of the TAPER consortium, a not-for-profit group of international researchers who conduct research on dose reduction and discontinuation of antipsychotics.**

This study protocol outlines a proposal to conduct PK modelling of olanzapine on both patients with AN and other patients taking the antipsychotic.

My main concerns pertain to the impression (given through their sample size calculation) that the authors are focussing more on the binary outcome of AN diagnosis, rather than the spectrum of body weight. The latter is far more likely to impact on PK parameters, and will be of far greater clinical use when recommending dosing etc.

The authors present a cursory overview of existing literature in their introduction, but further clarification and elaboration is required at points.

On the whole, methodology appears sound. The authors defend their pharmacokinetic sampling approach, which appears comprehensive in taking both DBS samples as well as venous (olanzapine DBS monitoring is, at least to my understanding, very novel and developing, so being able to cross-check this with venous samples is essential in my view).

The authors appropriately comment on the limitations of a cross-sectional design, especially for their secondary analytical elements.

In summary, this protocol still requires several revisions in my opinion before being publishable within BMJ Open. However, the study design and methodology overall in this protocol are credible and rigorous.

Line-by-line review feedback:

Abstract line 9 – 'pharmacokinetic' should be 'pharmacokinetics'

Abstract final line – 'with as' should be 'with a'?

Background line 4 – "As a result, AN carries one of the highest mortality rates…" – I would expand and clarify more as to why mortality rates are so high. E.g. this is not directly due to fear of gaining weight or distorted perception, but the extreme calorie restriction and low body weight. The way the sentences are currently phrased implies that the whole syndrome, rather than the biological aspects.

Background line 9 – "An observational study of 525 patients with AN showed that about 13% were using antipsychotics.(8)" – how many of these were considered treatment-resistant? The previous sentence implies that treatment-resistance is required before antipsychotics prescribed, but is this the case in clinical practice?

Background line 11 – is it worth commenting on the orexigenic properties of olanzapine? Could this contribute to its overall 'effectiveness' in terms of weight gain and reduced mortality? I note that this is referred to later in the background though.

Background line 18 – "To date, no studies have explored the pharmacokinetic properties of any psychotropic medications, including olanzapine, in individuals with AN". This is an interesting point, but AN in-and-of-itself will not affect pharmacokinetics. I would be more interested to know what the PK literature is in terms of assessing those with low body weight (which of course does not directly correlate with AN diagnosis and can result through multiple causes).

Background line 20 – "In cases of cachexia from other causes, changes in absorption, distribution, and metabolism have been documented. In AN, however, only absorption has been studied and found to be reduced." – explicitly for olanzapine? The references suggest not, and this is an important point to note.

Background paragraph 4 line 2 – BMI should be expanded (body mass index) at first mention.

Background paragraph 4 line 2 – "For these individuals, such an approach may result in subtherapeutic doses, potentially worsening their prognosis" – what is the evidence base for a 'therapeutic dose' in AN? Have efficacy studies been conducted to ascertain a dose-response (or even better a concentration-response) relationship?

Background paragraph 4 line 3 – I assume reference 23 is supporting the following statement: "Supporting this hypothesis, studies involving patients treated with olanzapine for conditions other than AN have shown that over one-third of patients deemed treatment-resistant to antipsychotics had subtherapeutic plasma levels of olanzapine"? If so, the reference should be moved to the end of this sentence.

Methods and design line 1 – "multicenter", but where? How many? International? Same region of same country? Further detail is required.

Figure 1 – the layout of this confused me, and I am unsure what benefit a figure provides over simply including this information in the text body of the manuscript. Instead, further detail should be provided in the figure, such as a summary on inclusion/exclusion criteria, what retrospective data will be collected, expanding the acronym 'DBS', and presenting the flowchart in a clearer stepwise manner, rather than boxes jutting off to the side without a clear reason or explanation.

Participants line 2 – why the upper age cut-off of 30 years?

Participants line 5 – For a PK study, reference must be made to patients being at steady-state and having not missed any doses within the past e.g. 5-7 days.

Sample size calculation paragraph 2 line 4 – "To account for impaired hepatic clearance in cachectic patients, we incorporated a 30% reduction in clearance, based on findings by Trobec et al." – what is the threshold for considering someone cachetic? This is not a binary outcome, and clearance reduction will surely reduce linearly with body weight reduction.

Sample size calculation paragraph 3 line 1 – "with body weights ranging from 31 to 54 kg and daily olanzapine doses between 2.5 and 12.5 mg" – why were these weights and dosages chosen? A taller person could have a weight closer to 60kg and still have a BMI which is categorised as underweight. Moreover, why was an upper limit of 12.5mg olanzapine used?

Sample size calculation paragraph 4 – Referring back to my comments regarding paragraph 2 of this section, the assumed reduction in clearance also raises questions for how the model was conducted – were the same range of body weights used for patients without AN? Artificially reducing clearance simply on the binary variable of AN diagnosis rather than the continuous measure of weight misses a key element of this calculation, and likely the ultimate conclusions of this study in terms of recommending dosing.

Pharmacokinetic sampling line 4 – "Microsamples will be collected at three different time points throughout the day" – when exactly? This is very important if you are purporting to be calculating Tmax, which to me feels a stretch when only measuring at three timepoints during a day.

Study parameters and assessments paragraph 2 line 3 – "The primary endpoint of this analysis is to evaluate the impact of AN on CL. This will allow us to compare pharmacokinetic parameters between AN and non-anorexic patients in order to optimized precision dosing in the AN population." – Similar to above, I would avoid using a binary categorisation of AN/not when devising modelling or dosing recommendations, as the impact is likely to be due to the more fluid measure of body weight.

Table 2 – I would argue that ECG is part of routine clinical care, especially for someone either with a low BMI/AN diagnosis, or for someone taking/being commenced on antipsychotic medication.

VERSION 1 - AUTHOR RESPONSE

Reviewer: 1
Dr. James O'Neill, University of Leeds School of Medicine, South West Yorkshire Partnership NHS Foundation Trust
Comments to the Author's:

This study protocol outlines a proposal to conduct PK modelling of olanzapine on both patients with AN and other patients taking the antipsychotic.

My main concerns pertain to the impression (given through their sample size calculation) that the Author's are focusing more on the binary outcome of AN diagnosis, rather than the spectrum of body weight. The latter is far more likely to impact on PK parameters, and will be of far greater clinical use when recommending dosing etc.

The Author's present a cursory overview of existing literature in their introduction, but further clarification and elaboration is required at points.

Author's response: Thank you for this important observation. We agree that body weight is likely an important determinant of PK parameters and that evaluating effects across the full weight spectrum may be more clinically informative than focusing solely on a binary diagnosis of AN. Therefore, in addition to investigating AN as a diagnostic category, we will evaluate the role of BMI as a continuous measure to assess the relationship between body size and pharmacokinetic parameters across the full range of body weights. In this analysis, however, AN was included as a dichotomous variable because we hypothesized that its impact may extend beyond low body weight alone. AN is associated with cachexia and a range of physiological alterations, including changes in gastrointestinal absorption, circulating protein concentrations, body composition, and other factors that may influence drug disposition and PK parameters independently of body size. By including AN as a diagnostic category, we aimed to explore whether disease-specific effects contribute beyond the established effect of body weight, which is already incorporated in the model used for the sample size calculation.

We have revised the text in the method to emphasize this point: *'…To evaluate the model's ability to detect a 30% reduction in clearance associated with AN, beyond the effect of body weight, that may influence drug disposition and pharmacokinetic parameters independently of body size..'*

In the primary outcome we already describe we will study both, AN and bodyweight, as mentioned in *"…we will investigate the effects of AN and body weight on these pharmacokinetic parameters."*

On the whole, methodology appears sound. The Author's defend their pharmacokinetic sampling approach, which appears comprehensive in taking both DBS samples as well as venous (olanzapine DBS monitoring is, at least to my understanding, very novel and developing, so being able to cross-check this with venous samples is essential in my view).

The Author's appropriately comment on the limitations of a cross-sectional design, especially for their secondary analytical elements.

In summary, this protocol still requires several revisions in my opinion before being publishable within BMJ Open. However, the study design and methodology overall in this protocol are credible and rigorous.

Line-by-line review feedback:

Abstract line 9 – 'pharmacokinetic' should be 'pharmacokinetics'

Author's response: Revision has been made in the text.

Abstract final line – 'with as' should be 'with a'?

Author's response: The discussion section in the abstract has been deleted as requested by the editor. Therefore, the spelling correction is no longer applicable in the revised manuscript.

Background line 4 – "As a result, AN carries one of the highest mortality rates…" – I would expand and clarify more as to why mortality rates are so high. E.g. this is not directly due to fear of gaining weight or distorted perception, but the extreme calorie restriction and low body weight. The way the sentences are currently phrased implies that the whole syndrome, rather than the biological aspects.

Author's response: Thank you for this suggestion. We have added the following clarification to the text: *'…owing to both the life-threatening physiological effects of chronic malnutrition and an increased risk of suicide.'*

Background line 9 – "An observational study of 525 patients with AN showed that about 13% were using antipsychotics.(8)" – how many of these were considered treatment-resistant? The previous sentence implies that treatment-resistance is required before antipsychotics prescribed, but is this the case in clinical practice?

Author's response: Thank you for this comment. Unfortunately, the proportion of patients with treatment-resistant AN in this cohort was not reported. While clinical experience suggests that approximately 20% of individuals with AN may have treatment-resistant illness, robust data on its prevalence remain limited. We agree that, in routine clinical practice, antipsychotics may be prescribed for a variety of reasons beyond treatment-resistant AN.

Revision in the text has been made: *'…Reflecting their use in routine clinical practice, an observational study of 525 individuals with AN reported that approximately 13% were receiving antipsychotic medication; however, the proportion of these patients with treatment resistance AN was not reported.(8)…'*

Background line 11 – is it worth commenting on the orexigenic properties of olanzapine? Could this contribute to its overall 'effectiveness' in terms of weight gain and reduced mortality? I note that this is referred to later in the background though.

Author's response: The efficacy of olanzapine is commonly attributed to its effects on obsessive-compulsive symptoms and psychological distress. Although olanzapine is associated with increased appetite and weight gain, the extent to which these effects contribute to its efficacy in anorexia nervosa remains debated. Importantly, olanzapine is typically prescribed to address psychological symptoms rather than specifically for its appetite-stimulating properties.

Background line 18 – "To date, no studies have explored the pharmacokinetic properties of any psychotropic medications, including olanzapine, in individuals with AN". This is an interesting point, but AN in-and-of-itself will not affect pharmacokinetics. I would be more interested to know what the PK literature is in terms of assessing those with low body weight (which of course does not directly correlate with AN diagnosis and can result through multiple causes).

Author's response: Thank you for this suggestion. We have revised the text as follow: *'… To date, no studies have explored the pharmacokinetic properties of any psychotropic medications, including olanzapine, in individuals with* ***extreme low body weights****….'*

Background line 20 – "In cases of cachexia from other causes, changes in absorption, distribution, and metabolism have been documented. In AN, however, only absorption has been studied and found to be reduced." – explicitly for olanzapine? The references suggest not, and this is an important point to note.

Author's response: Thank you for highlighting this. We apologize for the confusion. These data were not derived from studies of olanzapine. To clarify this point, we have revised the text to state: *'…In AN, however, absorption has only been investigated* ***for substances such as zinc, mannitol, and lactulose****….'*

Background paragraph 4 line 2 – BMI should be expanded (body mass index) at first mention.

Author's response: Revision has been made in the text.

Background paragraph 4 line 2 – "For these individuals, such an approach may result in subtherapeutic doses, potentially worsening their prognosis" – what is the evidence base for a 'therapeutic dose' in AN? Have efficacy studies been conducted to ascertain a dose-response (or even better a concentration-response) relationship?

Author's response: At the time this protocol was written, evidence linking olanzapine concentrations to treatment response in anorexia nervosa was scarce. Although the literature suggested a modest effect on weight gain, plasma concentrations were rarely measured, and no therapeutic concentration range had been established. A 2020 systematic review reported greater benefits at higher doses, but this finding was based on pooled heterogeneous studies rather than concentration-response analyses. (Copur et al, 2020)

These considerations has been added to the text: *'…For these individuals, such an approach may result in subtherapeutic doses, potentially worsening their prognosis. Although evidence linking olanzapine concentrations to treatment response in anorexia nervosa (AN) remains limited, higher doses have been associated with greater clinical benefit. However, this observation is based on pooled heterogeneous studies rather than formal concentration-response analyses….'*

Background paragraph 4 line 3 – I assume reference 23 is supporting the following statement: "Supporting this hypothesis, studies involving patients treated with olanzapine for conditions other than AN have shown that over one-third of patients deemed treatment-resistant to antipsychotics had subtherapeutic plasma levels of olanzapine"? If so, the reference should be moved to the end of this sentence.

Author's response: The reference has been moved to the end of the sentence.

Methods and design line 1 – "multicenter", but where? How many? International? Same region of same country? Further detail is required.

Author's response: Clarification has been added: *'…TORPEDO is a national, multicenter, cross-sectional cohort study designed to investigate the pharmacokinetics of olanzapine in patients with AN. In the Netherlands, participants will be recruited from five specialized psychiatric centers and two academic medical center….'*

Figure 1 – the layout of this confused me, and I am unsure what benefit a figure provides over simply including this information in the text body of the manuscript. Instead, further detail should be provided in the figure, such as a summary on inclusion/exclusion criteria, what retrospective data will be collected, expanding the acronym 'DBS', and presenting the flowchart in a clearer stepwise manner, rather than boxes jutting off to the side without a clear reason or explanation.

Author's response: Thank you for this suggestion. A stepwise explanation in text has been made as alternative to the flowchart.*'…Participants are screened for eligibility based on the study's inclusion and exclusion criteria. Those who meet the eligibility requirements and provide written informed consent are enrolled in the study. Following enrolment, retrospective data are collected from available records. Participants subsequently complete dried blood spot (DBS) day-curve sampling and self-report questionnaires as part of the study assessments. …'*

Participants line 2 – why the upper age cut-off of 30 years?

Author's response: Thank you for this important question. The upper age cut-off of 30 years was chosen to focus on a relatively homogeneous population in AN is most prevalent and to minimize potential confounding factors that may influence pharmacokinetic outcomes. By restricting inclusion to individuals aged 12 to 30 years, we aimed to reduce variability related to age-dependent physiological changes, comorbidities, and differences in body composition. Including older individuals could introduce additional factors, such as perimenopausal hormonal changes, age-related alterations in metabolism, and a higher burden of comorbid conditions, all of which may independently affect pharmacokinetic parameters. Therefore, this age range was selected to enable a clearer assessment of the effects of AN-related characteristics, including low body weight and malnutrition, on olanzapine pharmacokinetics.

Participants line 5 – For a PK study, reference must be made to patients being at steady-state and having not missed any doses within the past e.g. 5-7 days.

Author's response: Clarification in the text has been made: *'... All patients must be on a stable regimen of orally administered olanzapine, have achieved steady-state exposure, and have had no missed doses during the previous seven days….'*

Sample size calculation paragraph 2 line 4 – "To account for impaired hepatic clearance in cachectic patients, we incorporated a 30% reduction in clearance, based on findings by Trobec et al." – what is the threshold for considering someone cachectic? This is not a binary outcome, and clearance reduction will surely reduce linearly with body weight reduction.

Author's response: Thank you for this important observation. We agree that our previous reference to cachexia was not entirely accurate. While body weight is an important determinant of PK parameters and is already accounted for in the structural model, we hypothesize that AN may affect PK parameters beyond the effect of body weight alone. Available data from cachectic populations mainly point to a reduction in hepatic clearance. Although AN is distinct from cachexia, similar changes in hepatic drug metabolism may be expected due to shared features of severe undernutrition, but this remains to be confirmed empirically. Therefore, we included AN as a diagnostic category to explore potential disease-specific effects that may not be fully captured by body weight alone.

Revision in the text has been made: *'…To evaluate the model's ability to detect a 30% reduction in clearance associated with AN, beyond the effect of body weight, we simulated and re-estimated data using both the original model (including the AN effect on clearance) and an alternative model without this effect….'*

To account for the hypothesized impaired hepatic clearance in AN patients, we incorporated a 30% reduction in clearance, based on findings by Trobec et al. in cachectic patients.(14)

Sample size calculation paragraph 3 line 1 – "with body weights ranging from 31 to 54 kg and daily olanzapine doses between 2.5 and 12.5 mg" – why were these weights and dosages chosen? A taller person could have a weight closer to 60kg and still have a BMI which is categorised as underweight. Moreover, why was an upper limit of 12.5mg olanzapine used?

Author's response: Thank you for this observation. The body weight range of 31 to 54 kg was selected based on expert opinion and the characteristics of the patient population encountered in clinical practice. We acknowledge, however, that patients with AN may present with body weights outside this range. The upper olanzapine dose of 12.5 mg/day was chosen in accordance with the Dutch Kinderformularium recommendations for patients with AN. As the simulations were designed to reflect the dosing regimens and demographic characteristics of our target population, this value was used as the upper dosing limit.

Sample size calculation paragraph 4 – Referring back to my comments regarding paragraph 2 of this section, the assumed reduction in clearance also raises questions for how the model was conducted – were the same range of body weights used for patients without AN? Artificially reducing clearance simply on the binary variable of AN diagnosis rather than the continuous measure of weight misses a key element of this calculation, and likely the ultimate conclusions of this study in terms of recommending dosing.

Author's response: We agree that the planned covariate analysis should be described more clearly. In the population PK model, both body weight and AN diagnosis will be evaluated as potential covariates. Given the well-established influence of body weight on pharmacokinetic parameters, we anticipate that body weight will first explain a substantial proportion of the variability in clearance and/or volume of distribution. Subsequently, AN diagnosis will be assessed to determine whether it provides additional explanatory value beyond the effect of body weight alone, thereby capturing potential disease-specific effects associated with AN. We have revised the text accordingly and have also added AN diagnosis to the list of covariates evaluated in the covariate analysis.

For participants without AN, a higher body weight range was applied to reflect the characteristics of the non-AN population.

Pharmacokinetic sampling line 4 – "Microsamples will be collected at three different time points

throughout the day" – when exactly? This is very important if you are purporting to be calculating Tmax, which to me feels a stretch when only measuring at three timepoints during a day.

Author's response: Optimal sampling time points were identified at 4, 8, and 23 hours after dose administration. In clinical practice, sampling will be scheduled as close as possible to these time points while minimizing interference with the patient's normal daily activities, including sleep. Sampling times will be determined individually according to the dosing schedule and in consultation with the patient. Patients will be required to accurately document all dosing and blood sampling times.

The clarification has been added to the text: *'… In clinical practice, blood samples will be collected as close as possible to these target time points while minimizing disruption to patients' daily routines, including sleep. Sampling schedules will therefore be individualized based on the dosing regimen and determined in consultation with each patient. To ensure accurate pharmacokinetic assessment, patients will be instructed to record the exact times of both dose administration and blood sample collection….'*

Study parameters and assessments paragraph 2 line 3 – "The primary endpoint of this analysis is to evaluate the impact of AN on CL. This will allow us to compare pharmacokinetic parameters between AN and non-anorexic patients in order to optimized precision dosing in the AN population." – Similar to above, I would avoid using a binary categorisation of AN/not when devising modelling or dosing recommendations, as the impact is likely to be due to the more fluid measure of body weight.

Author's response: Please be referred to the explanation above, paragraph 2.

Table 2 – I would argue that ECG is part of routine clinical care, especially for someone either with a low BMI/AN diagnosis, or for someone taking/being commenced on antipsychotic medication.

Author's response: You raise an important point. While ECG monitoring is strongly recommended by current guidelines, it is not yet standard practice in all clinical settings. Therefore, ECG assessment is not considered a mandatory study procedure, and we no longer assume that ECG data will be routinely available for all participants. When available, ECG data obtained as part of routine clinical care will be collected retrospectively and included in the study results.

*** ***

COI statement(s):

Reviewer: 1

If you have selected 'Yes' above, please provide details of any competing interests.: I am a member of the TAPER consortium, a not-for-profit group of international researchers who conduct research on dose reduction and discontinuation of antipsychotics.

VERSION 2 - REVIEW

**Reviewer**	**1**
**Name**	**O'Neill, James**
**Affiliation**	**University of Leeds School of Medicine**
**Date**	**25-Aug-2026**
**COI**	

The authors have appropriately defended many of the key recommendations for revision.

I remain unconvinced that the psychiatric condition of AN in-and-of-itself results in different pharmacokinetic ramifications to, say, starvation and extreme low body weight through other causes. I struggle to see what “disease-specific effects” on pharmacokinetics could be referring to aside from cachexia, which of course is not restricted to AN alone. Therefore, greater acknowledgement of the fact that AN forms a sort of proxy measure of starvation should be made in my view.

In terms of reduced hepatic clearance due to cachexia, I would repeat my earlier concern that cachexia is not a binary variable, and will not result in a binary outcome of either no reduced clearance or a 30% reduction. Instead, this will no doubt be a more linear relationship between rate/quantity of food/fluid intake and clearance. For the purposes of a sample size calculation, this does not need to be addressed further in my opinion, but should be considered in the final devising of the pharmacokinetic model.

Introduction para 2 first sentence – clarify in greater deal what exactly is being referred to in terms of ‘effectiveness’.

I accept the author’s justification for an upper age limit of 30 years, but this rationale needs to be included in the manuscript as well as the rebuttal letter.

There also remain some minor grammatical areas to address:

Abstract line 7 – should read “pharmacokinetic”

Introduction para 1 final sentence – should read “treatment-resistant AN”.

Introduction para 4 line 2 – remove the ‘s’ after the parentheses

---

VERSION 2 - AUTHOR RESPONSE

Reviewer: 1
Dr. James O'Neill, University of Leeds School of Medicine, South West Yorkshire Partnership NHS Foundation Trust
Comments to the Authors:
The authors have appropriately defended many of the key recommendations for revision.

I remain unconvinced that the psychiatric condition of AN in-and-of-itself results in different pharmacokinetic ramifications to, say, starvation and extreme low body weight through other causes. I struggle to see what “disease-specific effects” on pharmacokinetics could be referring to aside from cachexia, which of course is not restricted to AN alone. Therefore, greater acknowledgement of the fact that AN forms a sort of proxy measure of starvation should be made in my view.

Author's response: We agree that starvation/cachexia will be the main reason for the effect we suspect. Starvation and severe undernutrition can affect hepatic enzyme activity, liver blood flow, protein synthesis, and energy-dependent metabolic processes. These changes may alter clearance of hepatically metabolized drugs, like olanzapine. Besides this, changes in protein binding can effect availability of drugs for metabolism. AN also substantially alters body composition, which can result in changes in volume of distribution as well. Delayed gastric emptying and gastrointestinal dysmotility are commonly seen in AN and may alter the rate and extent of oral drug absorption. In conclusion, we expect a small person of 40kg, with normal BMI would result in different olanzapine PK compared to a 40kg AN patient. We expect these changes will not be described adequately using bodyweight corrections alone. We agree this will probably not be a binary effect and will investigate several formulas based on BMI or other clinical parameters to describe this effect.

In terms of reduced hepatic clearance due to cachexia, I would repeat my earlier concern that cachexia is not a binary variable, and will not result in a binary outcome of either no reduced clearance or a 30% reduction. Instead, this will no doubt be a more linear relationship between rate/quantity of food/fluid intake and clearance. For the purposes of a sample size calculation, this does not need to be addressed further in my opinion, but should be considered in the final devising of the pharmacokinetic model.

Author's response: Thank you for this insightful comment. We agree that cachexia is a continuum rather than a binary condition and that any associated reduction in hepatic clearance is likely to vary proportionally with the severity of cachexia, including factors such as nutritional and fluid intake. As this assumption was included solely for the purposes of the sample size calculation, we have not expanded on it further in the current manuscript. However, we acknowledge the importance of capturing this relationship more realistically and will consider incorporating cachexia as a continuous predictor in the development of the final pharmacokinetic model.

Introduction para 2 first sentence – clarify in greater deal what exactly is being referred to in terms of 'effectiveness'.

Author's response: Additional clarification has been made.

'*Among all antipsychotics, olanzapine demonstrated the strongest evidence of effectiveness in the treatment of AN, most notably in promoting increases in body mass index over time.'*

I accept the author's justification for an upper age limit of 30 years, but this rationale needs to be included in the manuscript as well as the rebuttal letter.

Author's response: The rationale has been added to the methods and analysis – Participants section

'… Inclusion was restricted to individuals aged 12 to 30 years to minimize age-related variability and facilitate assessment of the effects of anorexia nervosa on olanzapine pharmacokinetics….'

There also remain some minor grammatical areas to address:
Abstract line 7 – should read "pharmacokinetic"
Introduction para 1 final sentence – should read "treatment-resistant AN".
Introduction para 4 line 2 – remove the 's' after the parentheses

Author's response: Thank you for your careful and thorough review. The requested changes have been made in the text.
