# Supplementary material for: Characterising the pharmacokinetics of olanzapine in patients with anorexia nervosa (TORPEDO): protocol for a multicentre cross-sectional study
Source: BMJ Open. 2026 Sep 15;16(9):e119486. doi: 10.1136/bmjopen-2026-119486 (PMC13583754; doi:10.1136/bmjopen-2026-119486)
Supplement: Supplementary file 2 [file bmjopen-16-9-s001.docx]

|  | **STUDY PERIOD** | |
| --- | --- | --- |
|  | **Enrolment** |  |
| **TIMEPOINT** | **T=0** | **T=1** |
| **ENROLMENT** | | |
| **Eligibility screen** | X |  |
| **Informed consent** | X |  |
| **ASSESSMENTS** | | |
| **DBS** |  | X |
| **DNA sampling** |  | X |
| **Demographics** | X | X |
| **Physician observations** | X | X |
| **Questionnaires** |  | X |
| **Laboratory measurements** |  | X |

**Table S1.** SPIRIT Figure of the timeline of TORPEDO
